# A Departure

**Published:** 1912-07

**Authors:** 


### EDITORIAL DEPARTMENT.

**DR. F. E. DANIEL, Editor** **DR. R. H. L. BIBB, Associate Editor**

### A Departure

There has been added to the features of the "Red Back" a department of especial interest to women, to be conducted by Mrs. F. E. Daniel, in which will appear each month, in addition to editorials, contributions by distinguished men and women whose names will be announced later, for the especial benefit of women.

Mrs. Daniel says, truly, "You doctors talk to each other through the journals and in meetings, and the very persons most interested in certain subjects never hear of them." It is proposed to put the "Red Back" into the hands of others than doctors; to have it reach the women, not only of doctors' families but of those of lay people; and it is hoped and believed that much good can thus be accomplished in sanitation, household and personal hygiene, and by teaching mothers how and what to teach their daughters—all tending to social uplift, health and happiness. We hope our doctors will take the "Red Back" home with them and aid us in this laudable undertaking. Mrs. D. is assured of the co-operation of leaders and authorities in this line, both men and women, lay and professional.

The "Red Back" becomes, also, the official organ of the State Society of Hygiene, and each month one or more papers will be contributed by the members of that Association. This will constitute another department, to be conducted by Drs. Duggan and Hull, President and Secretary of the Association.

The "Red Back" is on a new "boom."

